# Demographic and behavioral characteristics of non-sex worker females attending sexually transmitted disease clinics in Japan: a nationwide case-control study

**DOI:** 10.1186/1471-2458-10-106

**Published:** 2010-03-01

**Authors:** Masako Ono-Kihara, Tatsuya Sato, Hideko Kato, Sonia P Suguimoto-Watanabe, Saman Zamani, Masahiro Kihara

**Affiliations:** 1 Department of Global Health and Socio-epidemiology, Kyoto University School of Public Health, Yoshida-Konoe-cho, Sakyo-ku, Kyoto 606-8501, Japan; 2 Joint United Nations Programme on HIV/AIDS Collaborating Centre for Socio-epidemiological HIV Research, Yoshida-Konoe-cho, Sakyo-ku, Kyoto 606-8501, Japan; 3 Faculty of Medicine, Kyoto University School of Medicine, Yoshida-Konoe-cho, Sakyo-ku, Kyoto 606-8501, Japan; 4 Japan Foundation for AIDS Prevention, 1-3-12 Misaki-cho, Chiyoda-ku, Tokyo 101-0061, Japan

## Abstract

**Background:**

Although number of sexually transmitted infections (STIs) reported in STI surveillance increased rapidly for women in Japan during the 1990s, the sexual behavior of women potentially at risk of STI infection remains unknown.

**Methods:**

In order to determine the demographic and behavioral characteristics of non-sex worker (SW) females attending STI clinics, female attendees (n = 145), excluding SW, from nine clinics across Japan and female controls from the general population (n = 956), both aged 18-50 years, were compared using two data sets of nationwide sexual behavior surveys conducted in 1999.

**Results:**

Although the occupation-type and education level were unrelated to STI clinic attendance in multivariate analysis, non-SW females attending STI clinics were younger (adjusted odds ratios [AOR] = 0.94, 95%CI: 0.89, 0.99), and more likely to be unmarried (AOR = 4.11, 95% CI: 1.73, 9.77) than the controls from the general population. In the previous year, STI clinic attendees were more likely to have had multiple partnerships (AOR = 3.09, 95% CI: 1.42, 6.71) and unprotected vaginal sex with regular partners (AOR = 3.59, 95% CI: 1.49, 8.64), and tended to have had their first sexual intercourse at a younger age (AOR = 1.77, 95%CI: 0.89, 3.54) and more unprotected vaginal and/or oral sex with casual partners (AOR = 2.08, 95%CI: 0.75, 5.71). Identical sexual behavior patterns were observed between the female attendees with a current diagnosis of STI (n = 72) and those before diagnosis (n = 73) and between those with a past history of STI (n = 66) and those without (n = 79).

**Conclusion:**

These results indicate that not only multiple partnerships or unprotected sex with casual partners, but also unprotected vaginal sex within a regular partnership is prevalent among non-SW female STI clinic attendees. The identical sexual behavior patterns observed between female attendees with a current STI diagnosis and those without, and between those attendees with a past history of STI diagnosis and those without, indicate that the result are unlikely confounded with the cases of non-STI infection. This sexual behavior pattern may be predictive of STI infection among young Japanese women and could have contributed to the STI epidemic in women in Japan during the 1990s.

## Background

National sexually transmitted infection (STI) surveillance in Japan witnessed a rapid increase in the reported number of STIs among women, especially in non-viral STIs such as chlamydial and gonococcal infections, beginning in the mid-1990s and reaching peaks in 2002 in both genders [1,2]. In women, average annual numbers of reported cases per designated clinic or hospital increased from 10.0 in 1995 to 27.8 in 2002 for genital chlamydia and from 1.3 to 4.7 for gonorrhea over the same period. Although the reported numbers of chlamydial and gonococcal infections have shown some decline in recent years (in 2006 the average numbers of reported cases per designated clinic or hospital were 19.2 and 2.4 for genital chlamydia and gonorrhea, respectively), they still remain high and other types of STIs such as genital herpes, condyloma acuminatum and syphilis have continued to increase over the same period [3].

Surveillance provides useful information regarding trends in STIs. It shows that genital chlamydia and gonorrhea are the most common types of STI among female patients, and that patients of 30 years old or younger account for 66% of all female cases [4]. However, since the demographic information collected in the surveillance is limited to age, gender and residential area, questions remain about what kind of sexual behaviors in what subpopulations have led to the recent increases in STIs in Japan. Such information is vital for developing effective STI/HIV prevention programs. In a recent case-control study using the data sets of nationwide surveys conducted in 1999, we determined the STI infection risk profiles of male STI clinic attendees in Japan [5]. Using the same data sets, this study attempts to describe demographic and behavioral characteristics of non-sex worker (SW) females attending STI clinics in Japan to gain insight into the sexual behavior patterns that drove the STI epidemic among women during the 1990s and subsequently contribute to the development of effective STI/HIV prevention programs to avert such epidemics.

## Methods

### Study design

The study employed a case-control design in which cases and controls were selected from two data sets of sexual behavior surveys conducted in Japan in 1999. One was from a sample of STI clinic attendees and the other from a probability sample of the general population. Both studies were designed by the authors of this study (MOK, MK) using the same set of questions, other than those specific to each study.

### Sexual behavior survey of the general population

The sexual behavior survey of the general population was conducted during June-July of 1999 [6]. A sample of 5000 individuals, aged 18-59 years, was selected from the general population using a two-stage cluster sampling procedure. Briefly, the entire country was divided into 11 regions. Each region was further divided into five population density bands, yielding 57 strata. A total of 5000 samples (2559 males and 2441 females) were allocated to each stratum in proportion to the population size. Within each stratum, sampling wards were selected in a probability proportional to size using ward lists prepared for the census survey. Around 20 samples were drawn systematically from the residents' basic register or electoral register from each ward. Each subject was visited by trained staff, four times at most if absent, and asked to complete an anonymous self-administered questionnaire. To maximize the response rate, visits were arranged at the time and day most convenient for the subject, as identified during the multiple visits. 1762 males and 1800 females were sampled without replacement, yielding final response rates of 68.8% and 73.7%, respectively.

### Nationwide STI clinic survey

The nationwide STI clinic survey was conducted during July-September of 1999 [5]. STI clinic attendees were recruited from 21 private STI clinics, including 9 clinics that reported female attendees, in six large cities (Sapporo, Sendai, Tokyo, Osaka, Hiroshima, and Fukuoka) within six districts (Hokkaido, Tohoku, Kanto, Kansai, Chugoku, and Kyushu) of Japan. The clinics were recruited through consultation with local STI physicians' associations and chosen based on their proximity to the largest entertainment district in each city. Subjects were selected from attendees at the clinics who were currently diagnosed with STIs or before diagnosis displaying STI-related symptoms during the study period. STIs included chlamydial infection, gonorrhea, syphilis, non-chlamydial non-gonococcal urethritis, genital herpes, condyloma acuminatum, chancroid, phthirus pubis, and STI-related symptoms included unusual genital discharge (flow), sores, warts, burning with urination, and redness or itching around the genitals. Eligible attendees were consecutively recruited and asked to complete an anonymous, self-administered questionnaire in a waiting room. A total of 1119 subjects participated in the survey, yielding a final response rate of 84.9 percent (791 males, 304 females, 24 unknown gender). As the survey was anonymous, not all participants responded, and information from the clinics could not be linked to the survey, the distribution of the exact diagnosis of STIs and STI-related symptoms among participants was not determined.

### Integration of the data sets

The data sets in the two surveys were combined for female subjects who lived in the six districts mentioned above. Subjects who had sexual intercourse during the previous year, met age criteria (18-50 year old) and denied involvement in commercial sex were included in the analysis. The merged data set included information about age, gender, occupation, educational background, marital status, HIV/STI-related knowledge, age at first sex, number of sexual partners in the previous year, types of sexual partners (regular, casual, or commercially-related), and condom use with each type of sexual partner in the previous year or during their last sexual experience. For STI clinic attendees, information on the presence of a current diagnosis of STI and a past history of STI infection excluding current diagnosis was included for subgroup analysis.

### Sample characteristics

The control group was somewhat older than that found in the 2000 census data [7]. The proportion of subjects in the age group of 18-29, 30-39 and 40-50 was 25, 37, and 38 percent respectively for the control group, and 37, 30 and 33 percent respectively for the female census population. Controls were more likely to be married than the census population (78 percent vs. 58 percent), and were better educated (50 percent vs. 42 percent for at least a college/university education). Occupational patterns were similar between the populations. Regarding STI clinic attendees, only age was available for comparison with the 1999 national sentinel STI surveillance data [8]. STI cases in this study were slightly younger than the STI surveillance population. The proportion of subjects in the age group of 18-29, 30-39, 40-50, was 79, 18, and 3 percent respectively, for STI clinic attendees, and 70, 24 and 6 percent respectively, for the STI surveillance population.

### Ethical issues

In both surveys, verbal informed consent was obtained from participants. They were then asked to complete the questionnaire and return it in a sealed envelope, in person or by mail. This research study was approved by the Committee for Research on Human Subjects at Kyoto University in Japan.

### Statistical analysis

All statistical analyses were performed using SPSS for windows (version 12.0; SPSS Inc., Chicago, Illinois, USA). Bivariate analyses were performed to determine the association between STI clinic attendance and demographic and behavioral variables. Logistic regression was conducted to calculate adjusted odds ratio (AOR) and 95 percent confidence intervals (CI). Answers to HIV/STI knowledge questions were transformed into scores by giving 1 for a correct answer and 0 for an incorrect answer. Behavioral variables were combined to create variables that coded presence (= 1) or absence (= 0) of unprotected sex for each type of partner. These variables were compulsorily entered into a multivariate model, together with age at first sex, number of sexual partners in the previous year, and demographic variables, except for the variables of behaviors practiced by too few participants and those strongly interrelated. All statistical tests were two-tailed and results were considered significant when p < 0.05.

## Results

The study examined data relating to 145 STI clinic attendees and 956 controls. Subjects in the control group who reported having had an STI in the previous year (n = 16) were excluded from the study.

Table 1 compares the demographic characteristics and HIV/STI-related knowledge of the two groups. STI clinic attendees were much younger than the control group (average age 24.9 vs. 36.3, *p *< 0.001). There was significant difference in the type of occupation between the groups (*p *= 0.012), with more employed individuals and less housewives among the STI clinic attendees than among the controls. Marital status varied between the groups with 78% of the controls being married while only 15% of the STI clinic attendees were married (*p *< 0.001). Education level was almost equivalent between the groups, with about 50% of both the STI clinic attendees and controls having at least a college/university education. Average scores on HIV/STI-related knowledge were higher for STI clinic attendees than for controls (11.9 vs. 9.6, *p *< 0.001).

**Table 1:** Demographic profile of Japanese non-sex worker female STI clinic attendees compared with population-based female controls

	STI* clinic attendees (n = 145)		Population-based controls (n = 956)		
			
Characteristic	n	%	n	%	*p *value†
**Age at survey**					< 0.001§
18-19	19	13.1	18	1.9	
20-29	96	66.2	225	23.5	
30-39	26	17.9	353	36.9	
40-50	4	2.8	360	37.7	
Missing	0	0	0	0	
Mean(SD*)	25.0(5.9)		36.1(8.7)		
Median	23		36		
**Employment**					< 0.001
Self-employed	7	4.8	86	9.0	
Management	0	0	6	0.6	
Employee	88	60.7	467	48.8	
Unemployed, full time student	40	27.6	57	6.0	
Housewife	5	3.4	326	34.1	
Missing	5	3.4	14	1.5	
**Marital status**					< 0.001
Married	21	14.5	746	78.0	
Not married	123	84.8	201	21.0	
Missing	1	0.7	9	0.9	
**Education level**					0.43
High school or below	66	45.5	471	49.3	
College/university or above	79	54.5	482	50.4	
Missing	0	0	3	0.3	
**HIV/STI-related knowledge score**¶					<0.001§
Mean(SD)	11.9(3.0)		9.6(3.6)		
Median	12		10		

* STI, sexually transmitted infection; SD, standard deviation

†*p *values for chi-square test unless otherwise noted

¶Score for HIV/STI-related knowledge is the total number of 18 questions answered correctly.

§*P *values for Student's *t*-test

Table 2 compares sexual behavior characteristics between the groups. STI clinic attendees experienced their first sexual intercourse almost three years earlier than the controls. Also, there was a remarkable difference in the number of sexual partners in the previous year. While only 8% of the controls reported that they had multiple partners in the previous year, 44% of STI clinic attendees reported multiple partners in the previous year. 40% of STI clinic attendees reported having had casual partners in the previous year, compared to only about 4% of controls.

**Table 2:** Sexual behavior profile of Japanese non-sex worker female STI clinic attendees compared with population-based female controls

	STI* clinic attendees (n = 145)		Population-based controls (n = 956)		
			
Characteristic	n	%	n	%	*p *value†
**Age at first sexual intercourse **(years)					<0.001
<19	105	72.4	244	25.5	
19 or older	38	26.2	641	67.1	
Missing	2	1.4	71	7.4	
Mean(SD*)	17.6(2.7)		20.5(3.3)		
Median	17		20		
**No. of partners **(previous year)					<0.001
1	56	38.6	871	91.1	
2 or 3	33	22.8	65	6.8	
4 or more	30	20.7	12	1.3	
Missing	26	17.9	8	0.8	
**Type of sex partner **(previous year)					
Regular partner					0.011
Yes	139	95.9	942	98.5	
No	6	4.1	10	1.0	
Missing	0	0	4	0.4	
Casual Partner					<0.001
Yes	58	40.0	35	3.7	
No	86	59.3	908	95.0	
Missing	1	0.7	13	1.4	
**Sex with regular partners **(previous year)					
Had unprotected vaginal sex					
Yes	119	82.1	622	65.1	<0.001
No	17	11.7	268	28.0	
Missing	9	6.2	66	6.9	
Had unprotected oral sex					
Yes	122	84.1	524	54.8	<0.001
No	18	12.4	375	39.2	
Missing	5	3.4	57	6.0	
Had unprotected anal sex					
Yes	10	6.9	42	4.4	0.31
No	130	89.7	851	89.0	
Missing	5	3.4	63	6.6	
**Sex with casual partners **(previous year)					
Had unprotected vaginal sex					
Yes	44	30.3	20	2.1	<0.001
No	94	64.8	922	96.4	
Missing	7	4.8	14	1.5	
Had unprotected oral sex					
Yes	40	27.6	17	1.8	<0.001
No	97	66.9	925	96.8	
Missing	8	5.5	14	1.5	
Had unprotected anal sex					
Yes	0	0	1	0.1	1.00
No	138	95.2	941	98.4	
Missing	7	4.8	14	1.5	

* STI, sexually transmitted disease; SD, standard deviation

†*p *values for chi-square test unless otherwise mentioned

Significant difference was observed between the groups in the prevalence of unprotected sexual practice. While the proportion of STI clinic attendees who experienced unprotected vaginal and oral sex with regular partners was 82% and 84%, respectively, the figures were 65% and 55% respectively, among the controls. Also, while the proportion of STI clinic attendees who experienced unprotected vaginal and oral sex with casual partners were 30% and 28%, respectively, with the figures only 2% for both among the controls. The proportions of those having had anal intercourse with either regular or casual partners were low in both groups without statistical difference between them.

Subgroup analysis of the female STI clinic attendees having self-reported current STI diagnosis (n = 73) and those before diagnosis (n = 72) revealed that the sexual behavioral patterns were identical between the groups with *p*-values of Chi-square tests for group difference all ranging between 0.77-1.00, except for unprotected oral sex with regular partners (*p *= 0.16). Subgroup analysis of the female STI clinic attendees with a past history of STI diagnosis (n = 66) and those without (n = 79) yielded similar results.

Multivariate analysis was performed to evaluate the independent association of demographic and behavioral variables with STI clinic attendance (Table 3). While age at the time of the survey was entered into the model as a continuous variable, occupation, educational level, marital status, age at first sexual intercourse, and number of partners in the previous year were entered collectively into the model, together with other behavioral variables that represent the presence of unprotected sex with regular or casual partners, all as dichotomous variables. HIV/STI-related knowledge scores and unprotected anal sex with casual partners were excluded from the analysis. Variables representing unprotected oral and vaginal sex with casual partners were combined to create a single dichotomous variable that represents the presence or absence of unprotected oral and/or vaginal sex, since these variables were closely correlated (r = 0.79).

**Table 3:** Bivariate and multivariate analyses on the demographic and sex behavioral correlates of STI infection among non-sex worker Japanese women.

Characteristic	Crude odds ratio	95%CI*	Adjusted odds ratio†	95%CI
**Socio-demographic factors**				
Age (years)	0.83	0.80-0.86	0.94	0.89-0.99
Occupation				
Unemployed, full time student or housewife	0.69	0.47-1.01	0.61	0.30-1.25
Others	1.00		1.00	
Education				
High school education or less	0.86	0.60-1.21	0.76	0.39-1.48
University education or above	1.00		1.00	
Marital status				
Unmarried	21.74	13.34-35.40	4.11	1.73-9.77
Married	1.00		1.00	
**Behavioral factors**				
First sexual experience (years)				
≦18	7.26	4.87-10.82	1.77	0.89-3.54
≧19	1.00		1.00	
Number of sexual partners (previous year)				
≧2	12.72	8.29-19.54	3.09	1.42-6.71
1	1.00		1.00	
Sex with regular partners (previous year)				
Had unprotected vaginal sex				
Yes	3.02	1.78-5.11	3.59	1.49-8.64
No	1.00		1.00	
Had unprotected oral sex				
Yes	4.85	2.91-8.10	1.34	0.57-3.18
No	1.00		1.00	
Had unprotected anal sex				
Yes	1.56	0.76-3.18	0.83	0.23-2.95
No	1.00		1.00	
Sex with casual partners (previous year)				
Had unprotected vaginal and/or oral sex				
Yes	23.16	13.51-39.68	2.08	0.75-5.71
No	1.00		1.00	

* CI, confidence interval

†Odds ratio was adjusted by multiple logistic regression analysis for districts (Hokkaido/Tohoku, Kanto-Koshinetsu, Chubu/Kinki, Chugoku/Kyushu)

Results of the multivariate analysis showed that female STI clinic attendees were younger (AOR = 0.94, 95% CI: 0.89, 0.99) and more likely to be unmarried (AOR = 4.11, 95% CI: 1.73, 9.77), while educational and occupational categories showed no significant association with STI clinic attendance. Female STI clinic attendees were more likely to have had multiple partners in the previous year (AOR = 3.09, 95%CI: 1.42, 6.71), and have had unprotected vaginal sex with regular partners (AOR = 3.59, 95% CI: 1.49, 8.64). Though not statistically significant, they tended to have experienced their first sexual practice at younger ages (AOR = 1.77, 95% CI: 0.89, 3.54), and have more unprotected vaginal and/or oral sex with casual partners in the previous year than the controls (AOR = 2.08, 95% CI: 0.75, 5.71). In order to eliminate any confounding effects of age, the same analysis was performed in the groups of STI clinic attendees (n = 139) and controls (n = 139) that were exactly frequency matched for age using one year intervals from 18 to 50 years old. Marital status and the same set of behavioral variables were found to be associated with STI clinic attendance in similar magnitudes as in the original unmatched analysis with statistical significance except for the age at first sexual intercourse and unprotected vaginal and/or oral sex with casual partners that were associated at the *p*-value level of between 0.1- 0.2.

## Discussion

This is the first study to evaluate the sexual-behavior profile of Japanese, non-SW females attending STI clinics utilizing the data sets collected in 1999. Using population-based controls, rather than hospital-based controls that bring a risk of over-controlling [9], our study shows that female STI clinic attendees are more likely to be younger, unmarried, have unprotected vaginal sex with regular partners in the previous year, and have multiple sex partners in the previous year. They also tended to have their first experience of sexual intercourse at a younger age and have more unprotected vaginal and/or oral sex with casual partners. These results however cannot be immediately translated into the risks for STI infection because the results may be confounded by attendees with non-STI infections such as vulvaginal candidasis, bacterial vaginosis or urinary tract infection. Confounding of such cases may well be why our study found unprotected sex with a regular partner was generally high among our subjects. However, this is unlikely to be the case because identical sexual behavioral patterns were identified between female attendees with a current diagnosis of STI and those before diagnosis, and between those with and without a past history of STI diagnosis. It is, therefore, likely that these sexual behaviors are predictive of STI infection among young women in Japan and could have contributed to the STI epidemic in women which Japan witnessed during the 1990s.

Case-control or cross-sectional studies that assess the possible STI infection risk of women using population-based controls are limited. These include a British study that compared females who attended STI clinics in the previous year (n = 250) with those who did not (n = 9584) among the probability samples of the general population using the data set of the British National Surveys of Sexual Attitudes and Lifestyles (NATSAL) conducted in 1990 [10]. Another British study, using the 2000 NATSAL samples, also compared females who had STIs in the previous five years (n = 416) with those who had not (n = 5459) [11]. In the U.S., two population-based studies were performed in North Carolina; one study compared black women with a lifetime history of gonococcal infection (n = 27) with women without such histories (n = 120) [12]; and the other study compared women in a low-income neighborhood with herpes simplex type 2 infections (n = 534) with those who had no such infection (n = 1101) [13]. In China, a national population-based study was conducted in 1999-2000 comparing women testing positive for chlamydia (n = 41) with negative controls (n = 1194) [14]. Finally, in Slovenia, a national population-based study was performed in 2000 that compared women with a lifetime history of STI infection (n = 41) and those without (n = 737) [15]. Although there are other studies that attempt to assess the correlates of STI infection in females, they either do not include the results of multivariate analysis for women or lack information on sample size [9,16,17].

The results of our study are consistent with all of the studies cited above, indicating that multiple partnerships is a strong correlate with STI infection or STI clinic attendance, though the time frame of the question and the stratification of multiple partners varies between the studies. While our study and the China study adopted the previous one year as the time frame for the questions on sexual behaviors, lifetime or the previous five years were used in other studies. Similarly, while the number of partners was used as a dichotomous variable of one or more in our study, it was used as a dichotomous variable with different categorization, a continuous variable or polychotomous variables in other studies. Our findings that STI clinic attendees are more likely to be unmarried or experienced sex at an earlier age are also consistent with the results of some of these studies in STI patients or STI clinic attendees [9,10,12].

Our study, however, differs importantly from other studies in analytic strategy. Though types of partners, types of sex or condom use are usually introduced as separate variables in analysis, we structured the questions so that we could construct dichotomous variables that represent the presence or absence of unprotected sex in each type of sex (vaginal, oral or anal) with each type of partner (regular, casual or paid). This enabled us to more accurately evaluate the potential risk of sexual behaviors for STI infection, especially the sexual behavior with regular partners that has not been adequately addressed because regular partnerships are usually used as a reference for other types of partnerships. Our analysis clearly showed that unprotected vaginal sex with a regular partner is an independent correlate of STI clinic attendance or STI infection. About 60% of female STI clinic attendees in our study experienced sex only with regular partners in the previous year, suggesting that not only multiple partnerships or unprotected sex with casual partners, but also unprotected sex with regular partners may pose a risk of STI infection for young women in Japan. It may be important to note the difference in the type of regular partnership between STI clinic attendees and controls. While 78% of the regular partners for controls were husbands, 77% of the regular partners of STI clinic attendees were boyfriends, who may be potentially short-term, which is consistent with the increased number of partnerships for STI clinic attendees.

The risk of sexual transmission through a regular partnership has been suggested in a number of studies on STIs or HIV [18-21]. These studies are, however, either case studies or cross-sectional studies that only show the proportion of people who are monogamous or have only a regular partner. To our knowledge, our study is the first to quantitatively assess the possible risk of unprotected sex with regular partners among women. The China study introduced variables that represent the level of income or socialization of the male steady partner and showed that women with chlamydial infection are more likely to have steady partners with higher incomes and displaying frequent socialization [14]. Since 98% of women having chlamydial infection had only a steady partner, it was suggested that infection from a steady partner is the single most important risk factor for STI infection for women in China. In view of the importance of the prevention of STI among women, more evidence on the risk of regular partnerships should be accumulated.

It is interesting to interpret the findings of the present study in relation to those of our previous study that analyzed the demographic and sexual behavioral risk profile of male STI clinic attendees using the same data sets and adopting the same analytic strategy [5]. That study showed that male STI clinic attendees are more likely to be unmarried, have multiple partnerships in the previous year, have unprotected vaginal sex with regular partners, have unprotected vaginal and/or oral sex with casual partners, and unprotected vaginal and oral sex with paid partners in the previous year. These findings, together with the results of the present study, suggest that Japanese women may be at risk of STI infection not only through casual or multiple partnerships but also potentially through regular partnerships with men who have frequent genital and/or oral sexual contact with paid or casual partners. Japanese women, especially unmarried women, may be at a greater risk of STI infection from male partners who buy sex than women in other developed countries because it was shown in our previous study [5] that the proportion of men who paid women for sex was 62.0% of male STI patients and 10.5% of probability male controls, while it is only a few percent among the general male population in other developed countries [22-24].

The results of the present study should be interpreted with caution. Although the case-control design utilized here is pertinent for rare diseases such as STIs, the analytic value may be compromised compared with cross-sectional studies utilizing a representative sample with nested cases and controls. In the present study, STI cases were sampled from private clinics. This is because over 90% of medical institutions in Japan are privately operated and because almost all Japanese people are covered by medical insurance programs, which are applied equally to both private and public institutions. Though selection bias should be considered, important characteristics of the female STI clinic attendees in the current study are shared with the 16 women with STIs in the previous year who were excluded from the control group. Like the STI clinic attendees in the current study, these women were, though to a lesser extent, more likely to have had experienced sex earlier, had unprotected vaginal or oral sex with regular partners or with casual partners and had multiple sex partners in the previous year than the women who had no history of STI infection in the previous year. Among control subjects, although the response rate for our survey (73.7%) was similar to other general population sexual behavior surveys [24-27], our samples were more likely to be married and better educated compared to the census population as described in the Methods section. Since marital status, but not education level, was strongly associated with sexual behavior, this could have affected the results of the bivariate comparison. It is, however, unlikely to have affected the results of multivariate analysis because results were adjusted for both education level and marital status. The control group could have also been biased in that the highly sexually-active subpopulation may have avoided the survey. However, our experience with a nationwide survey of students from 30 universities in 1999 using a similar questionnaire showed little association between the answers to the questions related to sexual behaviors and response rates, which ranged between 16.4-100% [28]. It is also possible that other unmeasured factors could have confounded the results, although in an attempt to avoid this four demographic and four district variables were included in the analysis. Finally, limitations in the results also exists in the fact that our data are 10 years old, making the extrapolation of the findings into the current STI epidemic among women difficult. The present study, however, remains valid because it aimed to analyze the possible background of the STI epidemic among women during the 1990s and this is the only data set available in Japan for this purpose.

Despite the possible limitations, the results of this study are important in showing the possible STI risk profile of non-SW females in Japan for the first time. Together with the results of male STI clinic attendees in our previous analyses, the present results suggest that the epidemic of STIs in young men and women which Japan has experienced since the mid-1990s may have been driven by the sexual network that has expanded among the younger population, linking sex workers and casual and regular partners, and increased in intensity due to multiple partnerships and the prevalent practice of oral sex. These finding should be translated into prevention programs. Of particular importance will be the education campaign to inform the public of the possible risk contained in regular partnerships for both men and women that has been long neglected. Reducing unprotected sex with sex workers by men that may bring STIs into casual and regular partnerships is also important.

Finally, in view of the rapid cultural globalization, the message from the present study may extend to other Asian countries experiencing similar changes in the sexual norms and behavior of young people [29,30].

## Conclusion

In a case-control design using population-based controls, our study described demographic and behavioral characteristics of non-sex worker (SW) females attending STI clinics. The results suggested that not only casual sex or multiple partnerships, but also unprotected vaginal sex with regular partners are predictive of STI infection among the non-SW, female population in Japan. HIV/STI prevention programs should focus on both the risk of frequent casual partnerships and the possible risk from regular partnerships that has been long-neglected.

## Competing interests

The authors declare that they have no competing interests.

## Authors' contributions

All authors contributed to this study. MOK designed and conducted the field surveys and was responsible for the analytical design. TS analyzed data and drafted the manuscript. HK and SZ helped with the statistical analysis and revised the manuscript. SPSW assisted subgroup analyses and age-matched analysis MK guided the overall study procedure including the data analysis and drafting and revising of the manuscript. All authors read and approved the final manuscript.

## Pre-publication history

The pre-publication history for this paper can be accessed here:

http://www.biomedcentral.com/1471-2458/10/106/prepub
